# Platelet mitochondrial DNA methylation predicts future cardiovascular outcome in adults with overweight and obesity

**DOI:** 10.1186/s13148-020-00825-5

**Published:** 2020-02-17

**Authors:** Sarah Corsi, Simona Iodice, Luisella Vigna, Akin Cayir, John C. Mathers, Valentina Bollati, Hyang-Min Byun

**Affiliations:** 1 William Leech Building, Population Health Sciences Institute, Newcastle University, Newcastle upon Tyne, NE2 4HH UK; 2 EPIGET Lab, Department of Clinical Sciences and Community Health, Università degli Studi di Milano, via San Barnaba 8, 20122 Milan, Italy; 3 Department of Preventive Medicine, Occupational Health Unit, Fondazione IRCCS Ca’ Granda, Ospedale Maggiore Policlinico, Milan, Italy; 4 Vocational Health College, Canakkale Onsekiz Mart University, Canakkale, Turkey

**Keywords:** mtDNA, DNA methylation, Platelets, CVD, Obesity

## Abstract

**Background:**

The association between obesity and cardiovascular disease (CVD) is proven, but why some adults with obesity develop CVD while others remain disease-free is poorly understood. Here, we investigated whether mitochondrial DNA (mtDNA) methylation in platelets is altered prior to CVD development in a population of adults with overweight and obesity.

**Methods:**

We devised a nested case-control study of 200 adults with overweight or obesity who were CVD-free at baseline, of whom 84 developed CVD within 5 years, while 116 remained CVD-free. Platelet mtDNA was isolated from plasma samples at baseline, and mtDNA methylation was quantified in mitochondrially encoded cytochrome-C-oxidase I (*MT-CO1*; nt6797 and nt6807), II (*MT-CO2*; nt8113 and nt8117), and III (*MT-CO3*; nt9444 and nt9449); tRNA leucine 1 (*MT-TL1*; nt3247 and nt3254); D-loop (nt16383); tRNA phenylalanine (*MT-TF*; nt624); and light-strand-origin-of-replication (*MT-OLR*; nt5737, nt5740, and nt5743) by bisulfite-pyrosequencing. Logistic regression was used to estimate the contribution of mtDNA methylation to future CVD risk. ROC curve analysis was used to identify the optimal mtDNA methylation threshold for future CVD risk prediction. A model was generated incorporating methylation at three loci (score 0, 1, or 2 according to 0, 1, or 2–3 hypermethylated loci, respectively), adjusted for potential confounders, such as diastolic and systolic blood pressure, fasting blood glucose, and cholesterol ratio. mtDNA methylation at *MT-CO1* nt6807 (OR = 1.08, 95% CI 1.02–1.16; *P* = 0.014), *MT-CO3* nt9444 (OR = 1.22, 95% CI 1.02–1.46, *P* = 0.042), and *MT-TL1* nt3254 (OR = 1.30, 95% CI 1.05–1.61, *P* = 0.008) was higher at baseline in those who developed CVD by follow-up, compared with those who remained CVD-free. Combined use of the three loci significantly enhanced risk prediction, with hazard ratios of 1.38 (95% CI 0.68–2.78) and 2.68 (95% CI 1.41–5.08) for individuals with score 1 or 2, respectively (*P* = 0.003). Methylation at these sites was independent of conventional CVD risk factors, including inflammation markers, fasting blood glucose concentration, and blood pressure.

**Conclusions:**

Methylations of *MT-CO1*, *MT-CO3*, and *MT-TL1* are, together, strong predictors of future CVD incidence. Since methylation of these mtDNA domains was independent of conventional CVD risk factors, these markers may represent a novel intrinsic predictor of CVD risk in adults with overweight and obesity.

## Background

Cardiovascular disease (CVD) is the single largest cause of death and is responsible for approximately 30% of all deaths worldwide [1]. Overweight and obesity are risk factors for CVD, attributed to insulin resistance [2], inflammation [3–5], and the hyperaggregability of platelets [6]. Subsequently, inflammation markers, such as C-reactive protein (CRP) [7], uric acid (UA) [8, 9], and fibrinogen [10], are used for CVD risk prediction, as are markers of platelet activation such as lipoprotein-associated phospholipase A2 (Lp-PLA2) [11]. However, not everyone with obesity develops CVD, and the reasons why some individuals with obesity develop CVD while others remain CVD-free are poorly understood.

Mitochondrial dysfunction and damage have been implicated in obesity [12, 13] and CVD [14]. In particular, platelet mitochondria are important in maintaining thrombosis and hemostasis [15]. Intriguingly, platelets show hyperaggregability in adults with obesity and are unresponsive to anticoagulant treatment [6, 16]. Mitochondria contain a circular genome of approximately 17 kb in size with 37 genes encoding for proteins, ribosomal RNAs, and transfer RNAs related to oxidative phosphorylation. There is growing evidence for epigenetic regulation of mitochondrially encoded genes through DNA methylation, supported by the identification of DNA methyltransferase activity in mitochondria [17], and these epigenetic marks are altered in response to environmental exposures [18, 19] and in disease states such as cancer [20]. It has recently been demonstrated that mitochondrial DNA (mtDNA) in platelets is aberrantly methylated in CVD patients [21], but whether this precedes disease development is not known. Supporting the hypothesis that such epigenetic changes in the mitochondrial epigenome may be early events related to CVD development, nuclear DNA methylation patterns in the liver are known to be modified by obesity [22], while epigenetic analysis of blood samples predicts future CVD risk [23–26]. DNA methylation is not only modified in CVD patients [27, 28], but also with exposure to CVD risk factors [29–33]. Further, the associations between DNA methylation and CVD events are often stronger in individuals with pre-existing CVD risk markers, such as obesity [23, 34]. Therefore, we hypothesized that aberrant platelet mtDNA methylation occurs in at-risk individuals, such as adults with obesity, prior to developing CVD and may therefore serve as a biomarker of CVD risk.

Here, we tested this hypothesis in a nested case-control study investigating the utility of platelet mtDNA methylation to predict future CVD events in adults with overweight or obesity who were CVD-free at baseline.

## Results

### Characteristics of participants

**Fig. 1:**
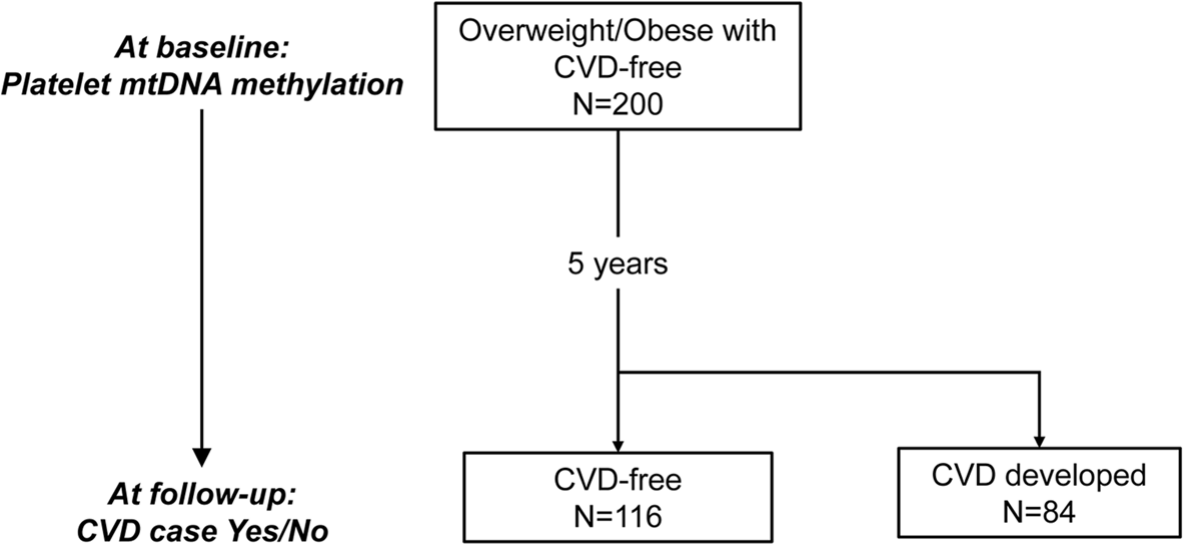
Study flow chart

The mean age of participants (*n* = 200) was 62 years (SD = 10), and 61% (*n* = 122) were female. The participants were overweight or obese (mean BMI = 35.5, SD = 5.1) and without CVD diagnosis at baseline. These participants were followed for up to 5 years, and the incidence of CVD was recorded (Fig. 1). At baseline, those participants who developed CVD during follow-up were BMI- and sex-matched to those who remained CVD-free. In addition, smoking status, education levels, blood pressure (systolic and diastolic (SBP and DBP)), fasting blood glucose, total cholesterol, high-density lipoprotein (HDL), low-density lipoprotein (LDL), and triglyceride levels at baseline were not significantly different by future CVD status (*P* > 0.05) (Table 1). Total cholesterol to HDL cholesterol ratio (TC/HDL) was lower at baseline in those who remained CVD-free compared to participants who developed CVD (CVD-free: mean = 3.7, SD = 1.1; CVD-developed: mean = 3.9, SD = 1.2; *P* = 0.039) (Table 1).

**Table 1:** Participant characteristics at baseline

Variable	All participants (*n* = 200)	CVD-free at the follow-up (*n* = 116)	CVD-developed at the follow-up (*n* = 84)	*P* value
Sex (*n*, %)				
Male	78 (39%)	44 (38%)	34 (40%)	0.716
Female	122 (61%)	72 (62%)	50 (60%)	
Age (mean, SD)	62.5, ± 10	61.7, ± 9.5	63.5, ± 10.6	0.210
BMI (mean, SD)	35.5, ± 5.1	35.4, ± 4.9	35.5, ± 5.4	0.936
BMI categorical (*n*, %)				
25.1–30.0 (overweight)	34 (17%)	22 (19%)	12 (14%)	0.762
30.1–34.9 (obesity I)	62 (31%)	33 (28%)	29 (35%)	
> 35.1 (obesity II and III)	104 (52%)	61 (53%)	43 (51%)	
Smoking status (*n*, %)				
Never	89 (45%)	53 (46%)	36 (43%)	0.859
Former	91 (46%)	50 (43%)	41 (49%)	
Current	19 (10%)	13 (11%)	6 (7%)	
Education, years of education (*n*, %)				
Primary school and other (< 5 years)	34 (17%)	18 (16%)	16 (19%)	0.297
Secondary school and high school (< 13 years)	129 (65%)	76 (66%)	53 (63%)	
University degree (> 14 years)	32 (16%)	21 (18%)	11 (13%)	
SBP, mmHg (mean, SD)	128.2, ± 13.7	129.1, ± 13.3	127, ± 14.1	0.268
DBP, mmHg (mean, SD)	78.9, ± 8.5	79.2, ± 8.5	78.4, ± 8.5	0.517
Fasting blood glucose, mmol/L (mean, SD)	5.9, ± 1.4	5.8, ± 1.4	6.0, ± 1.4	0.384
Total cholesterol, mg/dL (mean, SD)	206.6, ± 42.9	204.5, ± 42.4	209.5, ± 43.8	0.421
HDL cholesterol, mg/dL (mean, SD)	58.6, ± 15.0	60.0, ± 15.5	56.8, ± 14.3	0.141
LDL cholesterol, mg/dL (mean, SD)	128.3, ± 37.1	127.6, ± 36.0	129.1, ± 38.8	0.777
Triglyceride (TC), mg/dL (mean, SD)	126.4, ± 61.6	121.0, ± 57.6	133.7, ± 66.2	0.153
TC/HDL ratio (mean, SD)	3.7, ± 1.1	3.6, ± 0.9	3.9, ± 1.2	0.039
Framingham Risk Score, median (Q1, Q3)	18.2 (9.3, 28.9)	17.9 (9.6, 26.2)	18.3 (8.8, 30.5)	0.636
HeartScore, median (Q1, Q3)	2.0 (1.0, 3.0)	2.0 (1.0, 3.0)	2.0 (1.0, 4.0)	0.232
Medication usage (*n,* %)				
Not available	19 (9%)	6 (5%)	13 (16%)	0.039
Yes	46 (23%)	30 (26%)	16 (19%)	
No	135 (68%)	80 (69%)	55 (65%)	

### Platelet mtDNA methylation at baseline by future CVD development

**Fig. 2:**
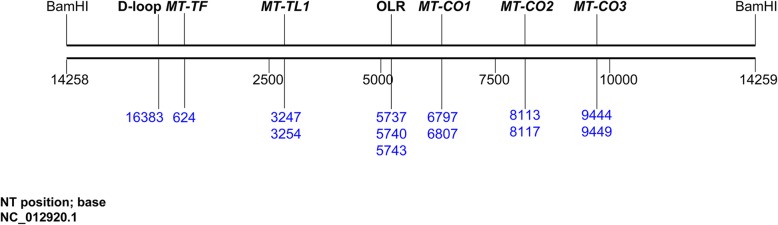
CpG locations within mitochondrial genome. Mitochondrial DNA was linearized using BamHI. The gene names and that of the displacement loop (D-loop), and the origin-of-replication of the light-strand (OLR) are annotated on the upper side. The nucleotide position of the CpG sites that have been analyzed is annotated below in blue text

**Fig. 3:**
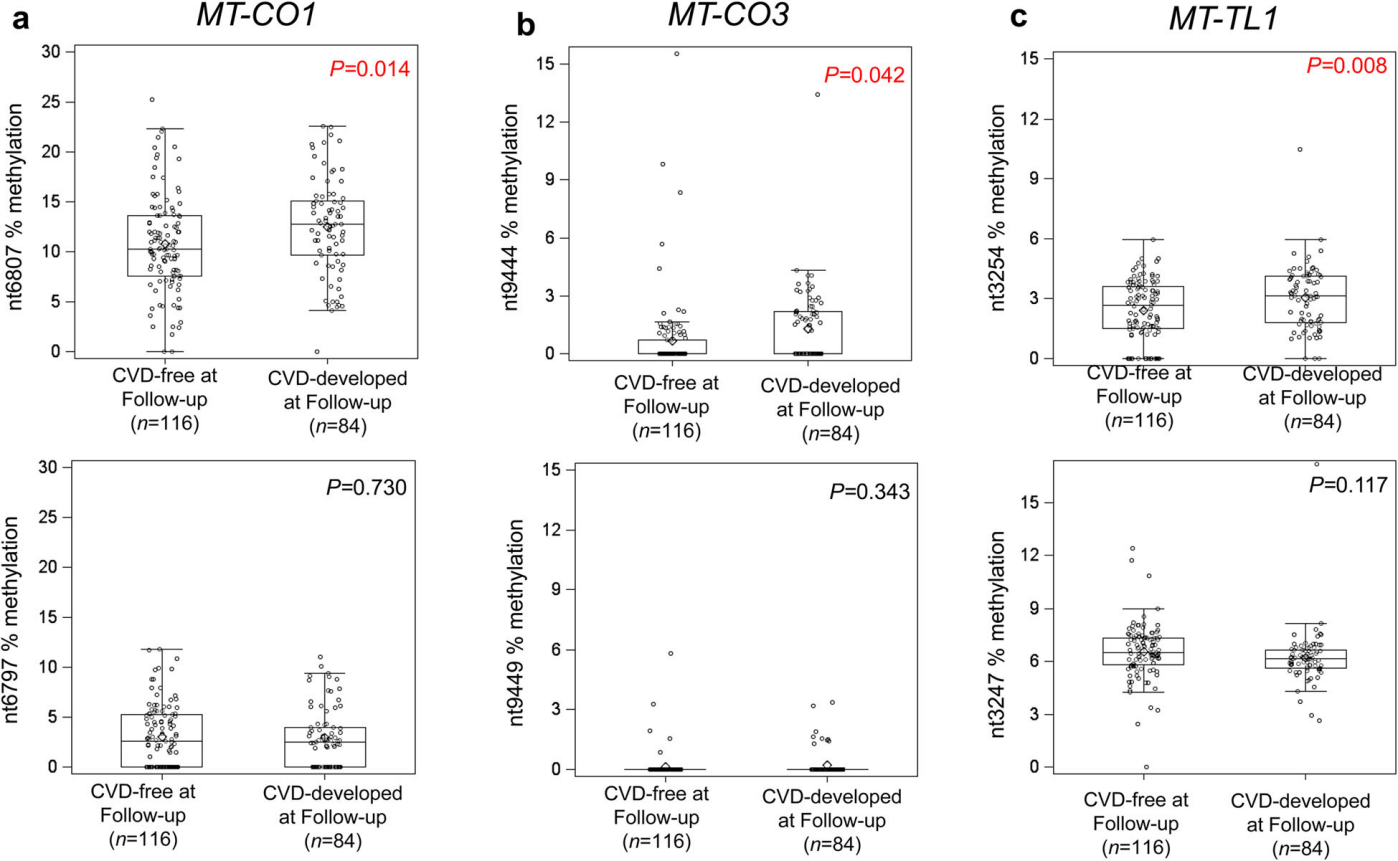
Distribution of mtDNA methylation at baseline among individuals who remained CVD-free and who develop CVD during follow-up. Methylation at two CpG positions for *MT-CO1* (**a**), *MT-CO3* (**b**), and *MT-TL1* (**c**), examined by pyrosequencing. The top panels report the CpG sites whose methylation significantly differs between the CDV-free and CVD-developed at follow-up. The *P* values were calculated by *t* test

We analyzed 13 CpG sites distributed within 7 mitochondrial genomic regions (Fig. 2). Methylation at baseline was lower in those participants who remained CVD-free compared with those who developed CVD during follow-up at nt6807 of *MT-CO1* (CVD-free: mean = 10.8 ± 4.8%; CVD-developed: mean = 12.5 ± 4.8%; *P* = 0.014), nt9444 of *MT-CO3* (CVD-free: mean = 0.7 ± 2%; CVD-developed: mean = 1.3 ± 1.9%; *P* = 0.042), and nt3254 of *MT-TL1* (CVD-free: mean = 2.4 ± 1.5%; CVD-developed: mean = 3.0 ± 1.6%; *P* = 0.008) (Fig. 3a–c). No significant differences in methylation were present for the other CpG sites measured.

**Fig. 4:**
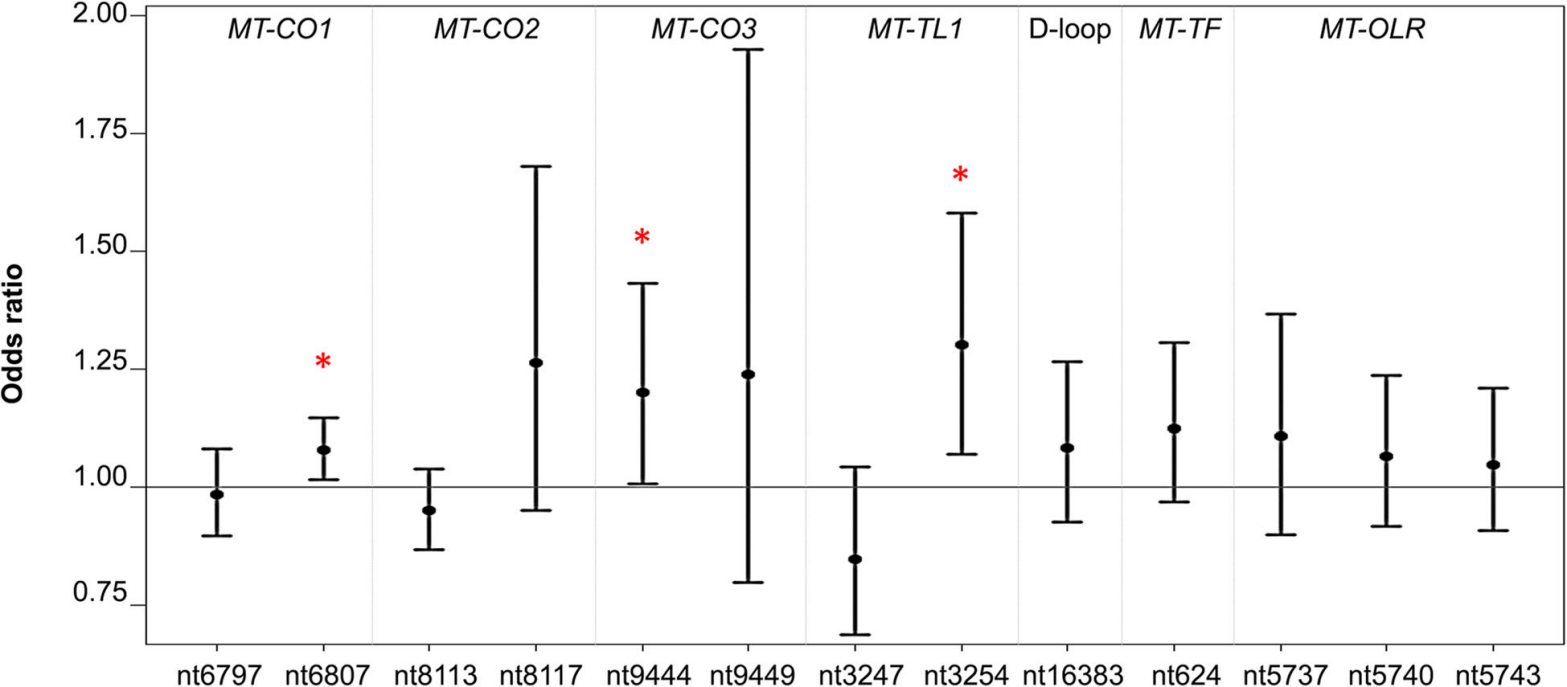
Odds ratios for the estimated contribution of each CpG site to future risk of CVD. The estimated effect of mtDNA methylation at each CpG site on the CVD outcome at follow-up, expressed as odds ratio (ORs) with 95% CI. Statistically significant positions are indicated by red asterisks. The analysis was performed by a multivariate logistic model adjusted for age, BMI, fasting blood glucose, cholesterol ratio, SBP, and DBP

We then examined methylation at the three loci in relation to the development of CVD during follow-up. The odds ratios (ORs) for developing CVD during follow-up were 1.08 (95% CI 1.02–1.16) for nt6807 of *MT-CO1*, 1.22 (95% CI 1.02–1.46) for nt9444 of *MT-CO3*, and 1.30 (95% CI 1.05–1.61) for nt3254 of *MT-TL1*, adjusted for age, BMI, fasting blood glucose, cholesterol ratio, SBP, and DBP (Fig. 4). Logistic regression demonstrated that there were no significant associations between mtDNA methylation of *MT-CO1*, *MT-CO3*, and *MT-TL1* and conventional CVD risk biomarkers at the baseline, including insulin resistance (HOMA-IR), age, cholesterol level, serum uric acid, and BMI (Table S1).

### Utility of platelet mtDNA methylation to predict CVD risk

**Fig. 5:**
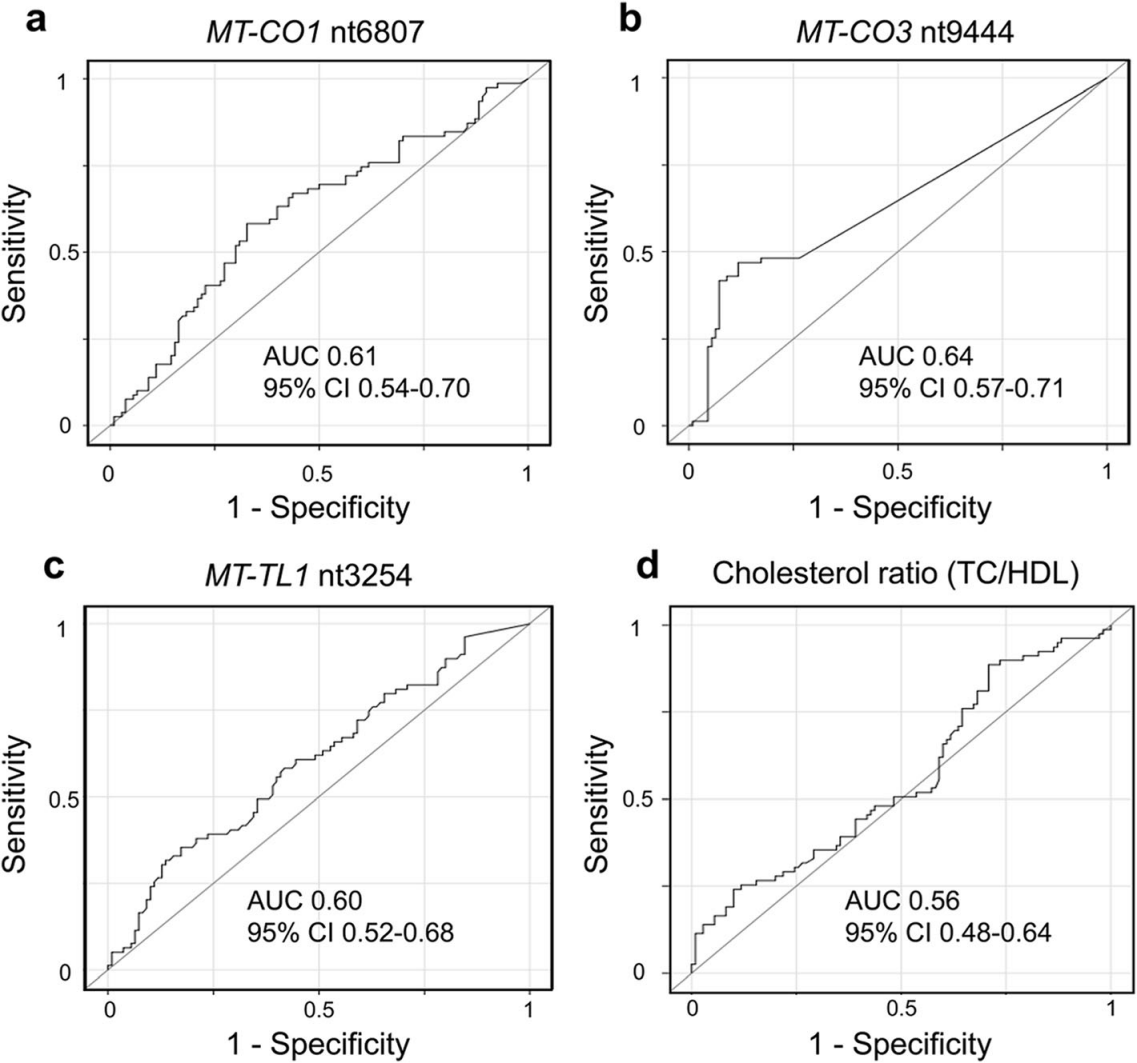
ROC curves for methylation at three loci and cholesterol ratio for prediction of CVD outcome. Discrimination ability of methylation at three CpG sites (*MT-CO1* nt6807, *MT-CO3* nt9444, and *MT-TL1* nt3254) (**a**–**c**) and the cholesterol ratio (TC/HDL) (**d**) to predict CVD incidence within 5 years of baseline. Area under the ROC curve (AUC) and 95% CI values are annotated

Receiver-operating characteristic (ROC) curves were generated to determine the optimal threshold of mtDNA methylation (%) for each CpG site at baseline to discriminate between CVD-free and CVD-developed individuals at follow-up (Fig. 5). Thresholds of 12% for *MT-CO1* nt6807 (*P* = 0.049), 1.5% for *MT-CO3* nt9444 (*P* = 0.001), and 3% for *MT-TL1* nt3254 (*P* = 0.22) yielded maximum discrimination between CVD-free and CVD-developed participants (Table 2 (a)). TC/HDL cholesterol, which differed between groups at baseline, was not a predictor of CVD risk during follow-up (*P* = 0.38) (Fig. 5d, Table 2 (a)).

**Table 2:** MtDNA methylation thresholds for each CpG site and score for predicting CVD outcome

a. Threshold for each CpG site						
	Methylation	Median survival time (months)^*^	At risk	CVD during follow-up	CVD free	Log-rank *P* value
All patients		43.8	200	84	116	
*MT-CO1* nt6809 (% methylation)	< 12.0	47.5	114	35	79	**0.049**
	≥ 12.0	38.3	83	47	36	
*MT-CO3* nt9444 (% methylation)	< 1.5	47.0	146	44	102	**0.001**
	≥ 1.5	33.0	51	38	13	
*MT-TL1* nt3254 (% methylation)	< 3.0	45.7	105	37	68	0.22
	≥ 3.0	42.1	94	46	48	
Cholesterol ratio	< 3.5	42.1	102	43	59	0.38
	≥ 3.5	45.3	94	41	53	
b. Score for predicting the CVD outcome						
Score**	Median survival time (months)	At risk	CVD during follow-up	CVD-free	% CVD-developed at follow-up	Log-rank *P* value
0	~ 60	61	13	48	21%	**0.003**
1	54.8	63	21	42	33%	
2	35.1	69	45	24	65%	

(a) MtDNA methylation thresholds for each CpG site and outcomes of survival analysis. Survival analysis for the participants stratified according to the methylation score at individual loci (*MT-CO1*, *MT-CO3*, and *MT-TL1*) and to the cholesterol ratio. (b) Score to predict future CVD events based on methylation at *MT-CO1* nt6809, *MT-CO3* nt9444, and *MT-TL1* nt3254.

*Median: time in months without-CVD

**Participants with score 2 (two or three CpG sites with methylation above the thresholds) had a lower median time without-CVD (35.1 months) than the participants with score 1 (54.8 months) and score 0 (the median survival time is not reached). This analysis was performed on a total of 193 participants, for whom the methylation percentage of all the three genes was available

**Fig. 6:**
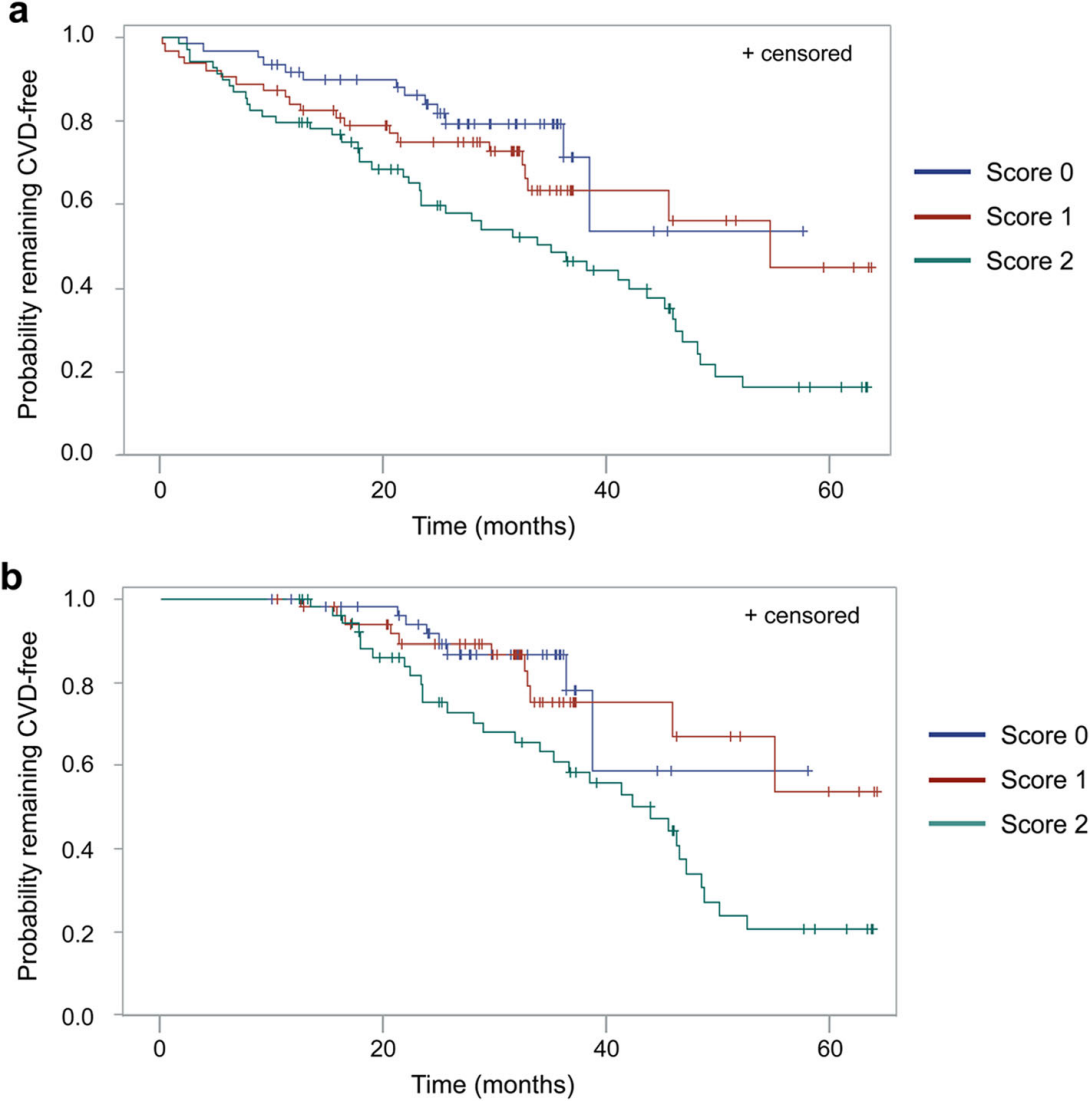
The Kaplan-Meier curves for probability of remaining CVD-free by methylation score. **a** Time CVD-free from baseline (months) among participants categorized by methylation at *MT-CO1* nt6807, *MT-CO3* nt9444, and *MT-TL1* nt3254. **b** Sensitivity analysis in which all participants who developed CVD within 1 year of baseline were excluded

The threshold values that maximized sensitivity and specificity to predict CVD risk were used to create dichotomous variables “methylation level above the threshold” and “methylation level below the threshold” for each of the significant CpG sites within *MT-CO1*, *MT-CO3*, and *MT-TL1.* Using these values, overall scores were calculated for each individual participant as follows: methylation not above the thresholds at any of the three loci (score 0), methylation above the threshold at any one locus (score 1), and methylation above the threshold at any two or all three loci (score 2) (Table 2 (b)). Compared with score 0, the hazard ratio (HR) for developing CVD for score 1 was 1.38 (95% CI, 0.68–2.78) and for score 2 was 2.68 (95% CI, 1.41–5.08) (Fig. 6a). During follow-up, 65% of the individuals with score 2 developed CVD, while only 21% of individuals with score 0 developed CVD (Fig. 6a and Table 2 (b)). Participants with score 2 had a lower median time without-CVD (35.1 months) than participants with score 1 (54.8 months). More than half of the participants with score 0 were CVD-free at the end of the follow-up period.

### Comparison with existing risk prediction models

Conventional CVD risk prediction scores were calculated at baseline using the Framingham Risk Score [35] and the European HeartScore [36]. These scores were not different between those who remained CVD-free and the CVD-developed group (*P* = 0.636 and *P* = 0.232, respectively), demonstrating the potential utility of mtDNA methylation as a predictor of CVD development.

### Sensitivity analysis

A sensitivity analysis was performed by excluding participants who developed CVD within a year from baseline, but this did not change the relationships previously observed (Fig. 6b). The HR for those who scored 2 remained significantly higher than those who scored 1 (HR = 2.17, 95% CI 1.06–4.47) and was even higher in comparison with those who scored 0 (HR = 2.53, 95% CI 1.12–5.72) (Fig. 6b). An additional sensitivity analysis was performed by stratifying the CVD cases into “Mild,” such as hypertension (*n* = 51), and “Severe” events, such as ischemic heart diseases (*n* = 33) (Table S2). The model was tested in the Mild subgroup and showed that the mtDNA methylation score was a significant (*P* < 0.001) predictor of future risk of developing CVD. The HR for those who scored 2 was significantly higher than for those scored 1 (HR = 2.27, 95% CI 1.13–4.44, *P* = 0.021) and those who scored 0 (HR = 4.34, 95% CI 1.76–10.73, *P* < 0.002). No such relationships were apparent in the Severe subgroup of CVD events (*n* = 33), due to lack of power (*P* = 0.086) (data not shown).

## Discussion

To the best of our knowledge, this is the first study investigating platelet mtDNA methylation in relation to the future development of CVD. In this nested case-control study of 200 adults with overweight and obesity, higher mtDNA methylation at three loci (*MT-CO1* nt6807, *MT-CO3* nt9444, and *MT-TL1* nt3254) in platelets was associated with higher risk of developing CVD within 5 years. Further, participants with score 2 (high methylation at two or three loci) developed CVD significantly sooner than the participants with score 1 and score 0. Thus, mtDNA methylation at the three loci may be a novel predictive biomarker for the future risk of developing CVD.

We have previously demonstrated changes in the mitochondrial epigenome among individuals with CVD, including hypermethylation of *MT-CO1*, *MT-CO3*, and *MT-TL1* [21]. Further, we have shown that mtDNA methylation modifies the effect of particulate matter exposure and heart rate variability, a prognostic marker of CVD [37]. We have built on our previous work to demonstrate that mtDNA methylation may serve as a predictor of CVD risk among individuals with overweight and obesity. However, the field remains at a nascent stage, with little understanding of the mechanisms underpinning how mtDNA methylation levels may be implicated in the etiology of CVD and/or platelet activation. Recently, it has been demonstrated that mtDNA methylation regulates expression of mitochondrial-derived peptides (MDP) with cytoprotective function [38] suggesting that mtDNA methylation level may be indicative of the overall stress to which the cell is exposed. Additionally, in vitro studies have shown that the presence of 5-methylcytosine can alter mitochondrial transcription factor (TFAM) binding and transcription initiation [39].

MtDNA methylation levels in blood are associated with blood pressure and heart rate variability in individuals with CVD-related environmental and occupational exposures [18, 19, 37, 40]. However, in platelets, we did not find any association between mtDNA methylation level and the most common CVD risk factors including age, BMI, blood pressure, blood glucose concentration, cholesterol, and uric acid in individuals with overweight and obesity. Therefore, our study supports the idea that altered mtDNA methylation in platelets precedes the development of CVD, and may serve as a non-invasive, easy-to-access biomarker to distinguish individuals with higher CVD risk. Adults with overweight or obesity may, therefore, benefit from identification to facilitate early primary prevention and monitoring to reduce their personal risk of CVD.

We observed low levels of mtDNA methylation in these participants and subtle, but detectable, differences between individuals who developed CVD during follow-up and those who remained CVD-free. Such subtle changes in methylation are not confined to the mitochondrial epigenome, as changes in methylation of < 5% are frequently reported in aging, in response to environmental exposures [41], and during disease initiation [42]. It is not known whether these small changes in DNA methylation reflect changes in gene expression. Regardless, they may serve as a biomarker of a cascade of other biological reactions [43–45], such as MDP regulation [38].

Our study has limitations that merit consideration. The outcome in our study was diagnosis of any of a heterogeneous group of CVDs that ranged from mild (e.g., hypertension) to more severe events. Our model remained strong in predicting the “mild” CVD events, but the lack of statistical power prevented examination of its ability to predict more “severe” cases. Further, replication of our findings is imperative. Such a validation would require access to data and samples from a cohort that had collected plasma or platelets and had follow-up data on CVD incidence as part of a prospective study of individuals with overweight and obesity. We utilized hospital discharge records, which are widely used for collection of data regarding clinical diagnoses (e.g., for Italian healthcare administrative databases and the WHO’s European Health Information Gateway for classification), but which can potentially under- or overestimate the number of cases. The use of thoroughly validated administrative databases may strengthen future studies. We attempted a partial validation by dividing the population of 200 individuals into a test set (*n* = 150) and a validation set (*n* = 50) with the same proportions of CVD-free and CVD-developed at follow-up participants in both, which showed that the mtDNA methylation markers predicted CVD risk in both the test (*P* = 0.045) and validation sets (*P* = 0.034). Finally, as most of the participants were Caucasian, additional studies are needed to validate these findings in individuals with different ethnicities.

In conclusion, we have demonstrated that mtDNA methylation of *MT-CO1*, *MT-CO3*, and *MT-TL1* in platelets from adults with overweight and obesity may predict CVD risk during the following 5 years. Our findings require confirmation in a larger, independent study.

## Methods

### Study design and sample selection

We utilized plasma samples and clinical data from the Susceptibility to Particle Health Effects, miRNAs and Exosomes (SPHERE) study in which 2000 participants with overweight (25 < BMI < 30 kg/m^2^) and obesity (BMI ≥ 30 kg/m^2^) were recruited in Milan, Italy [46]. We designed a prospective nested case-control study using samples and data from 200 participants within the SPHERE study without previous hospitalization for CVD at the time of enrolment (baseline; *n* = 200) for whom follow-up data for up to 5 years (median = 27 months) were available. For those who developed CVD, the follow-up stopped after the first CVD diagnosis; for those who remained CVD-free, the follow-up lasted until the last update from the Italian National Health Service. We selected 84 individuals who developed CVD in the follow-up period, and these were sex- and BMI-matched with 116 individuals who remained CVD-free. The demographic and clinical characteristics of these participants are summarized in Table 1. Ethical approval was provided by the Institutional Review Board, Fondazione IRCCS Cà Granda Ospedale Maggiore Policlinico at University of Milan. The ethnicity of the SPHERE study participants was predominantly Caucasian (95.8% of cases) [46].

### Assessment of CVD risk at baseline and CVD events at follow-up

To estimate individual CVD risk at baseline, we calculated the Framingham Risk Score which uses information on sex, age, SBP, treatment for hypertension, smoking, type 2 diabetes, HDL, and total cholesterol [35]. In addition, we used the HeartScore to predict the incidence of fatal CVD within 10 years [36, 47] using age, sex, SBP, cholesterol, HDL cholesterol, BMI, and smoking status.

Details of CVD events were obtained from the hospital discharge registry of the Italian National Health Service. A CVD event was defined as any principal or any 1 of 5 secondary diagnosis of diseases of the circulatory system (3-digit ICD-9-CM codes from 390 to 459) [48]. A detailed list of the CVD events and antihypertensive medications of the participants by follow-up is summarized in the Supplementary Material (Table S2 and S3). Briefly, 47 participants (56%) were diagnosed with hypertension and 37 participants (44%) were diagnosed with other forms of CVD including atrial fibrillation and atherosclerosis (Table S2).

### Platelet mtDNA preparation and DNA methylation measurement

Plasma samples from 200 participants were used to isolate platelet mtDNA as described previously [21]. Briefly, platelet pellets obtained by centrifugation of 200 μL plasma at 1400×*g* were treated with DNaseI (30 U, ROCHE) to eliminate cell-free nuclear DNA containing nuclear mitochondrial DNA segments (NUMTs). The EZ DNA Methylation Direct kit (Zymo Research) was used for extraction and bisulfite conversion of mtDNA simultaneously. To maximize bisulfite conversion efficiency [49], mtDNA was linearized with BamHI (NEB) following proteinase K treatment. The bisulfite-converted mtDNA (20 μL) was stored at − 80 °C until analysis.

Bisulfite-PCR reactions were performed using 1 μL of bisulfite-converted mtDNA, 9 μL water, 12 μL Hot-Start GoTaq® DNA Polymerase (Promega), 1 μL forward primer (10 pmole), and 1 μL reverse biotin-labeled primer (10 pmole). We investigated seven regions: mitochondrially encoded cytochrome-C-oxidases I, II, and III (*MT-CO1*, *MT-CO2*, and *MT-CO3*); mitochondrially encoded tRNA leucine 1 (*MT-TL1*) and tRNA phenylalanine (*MT-TF*); D-loop; and mitochondrially encoded light-strand origin-of-replication (*MT-OLR*). DNA methylation was measured at two CpG sites within *MT-CO1* (nucleotide (nt) positions 6797 and 6807), *MT-CO2* (nt8113 and nt8117), *MT-CO3* (nt9444 and nt9449), and *MT-TL1* (nt3247 and nt3254); one CpG site within D-loop (nt16383) and *MT-TF* (nt624); and three within *MT-OLR* (nt5737, nt5740, and nt5743) (Table S4). The provided mtDNA sequences and the nucleotide positions are based on NCBI reference sequence NC_012920.1. Amplified mtDNA PCR products were then used for pyrosequencing reactions (PyroMark Q96 ID, QIAGEN) as described elsewhere [18, 19, 21, 50]. Each sample was analyzed in duplicate (Pearson’s correlation coefficient = 0.74 for technical replicates; coefficient of variation 12.5%), and the mean of replicates was used for further analysis. The correlations between methylation at different CpG sites within each gene were low (Table S5), and we therefore treated each CpG as a separate data point.

### Statistical analysis

For normally distributed demographic and clinical characteristics and for DNA methylation levels, data are expressed as mean and standard deviation, otherwise by median and range. Frequencies and percentages were calculated for categorical variables. Data for CVD-free and CVD-developed participants at follow-up were compared using the *χ*^2^ test for categorical data and Student’s *t* test for continuous variables. Multivariate logistic regression, adjusted for age, BMI, fasting blood glucose, cholesterol ratio (TC/HDL), SBP, and DBP, was performed to investigate the association between DNA methylation at each locus (CpG site) and the risk of CVD during follow-up. Estimated effects were reported as ORs and 95% confidence intervals (CI) associated with an increase in 5-methylcytosine (5mC) at each locus.

ROC curves were generated to evaluate the diagnostic ability of the cholesterol ratio and mtDNA loci to distinguish between participants who were CVD-free and those in whom CVD-developed at follow-up. The optimum threshold was selected by the Youden Index as the one that maximized sensitivity (SE) + specificity (SP) − 1. The area under the ROC curve (AUC) and corresponding 95% CI, SE, SP, and threshold were reported for cholesterol ratio and for the *MT-CO1* nt6807, *MT-CO3* nt9444, and *MT-TL1* nt3254 positions. For each CpG site, a dichotomous variable was created viz. “methylation level above the threshold” for the specific locus and “methylation level below the threshold.” In addition, we tested the utility of a score built as the sum of the index value (0, 1) for each locus (*MT-CO1* nt6807, *MT-CO3* nt9444, and *MT-TL1* nt3254) in predicting CVD. The score has three categories: none of three loci display mtDNA methylation above threshold (score 0), any one of the three loci has mtDNA methylation above threshold (score 1), and any two or all three loci display mtDNA methylation threshold (score 2).

The Kaplan-Meier survival curves and log-rank tests were calculated by stratifying CVD cases by each locus below or above the methylation threshold. To evaluate the independent prognostic value of each single locus and of their combination on future CVD cases, we calculated hazard ratios (HRs) with Cox multivariable regression models adjusted for DBP, SBP, fasting blood glucose concentration, and cholesterol ratio. The Cox multivariable regression was performed on a total of 193 participants, for whom the methylation percentage of all the three genes was available. The same model was used to evaluate the prognostic value of cholesterol ratio, when evaluated as the predictor. The assumption of proportional hazard was checked with the log [log(survival)] plot and by the time-dependent covariate test. Cox multivariable regression models were also used to evaluate the potential prognostic value of the Framingham Risk Score and of the European HeartScore on CVD risk.

A sensitivity analysis was performed by excluding the participants who developed CVD within a year from baseline in all Cox multivariable regression models. An additional sensitivity analysis was performed by stratifying the CVD cases into “Mild,” such as hypertension (*n* = 51), and “Severe” events, such as ischemic heart diseases (*n* = 33). However, the Severe event (*n* = 33) category did not provide enough power to be tested reliably (data not shown). All reported *P* values were two-tailed, and those less than 0.05 were considered statistically significant. Statistical analyses were performed with SAS software, version 9.4.

## Supplementary information

**Additional file 1 Table S1.** Multivariate analysis for the association between potential risk factors and mitochondrial DNA methylation. **Table S2.** Diagnostic and procedure codes for CVD events at Follow-up. **Table S3.** List of antihypertensive medication. **Table S4.** PCR primers and pyrosequencing assays used to analyze mtDNA methylation. **Table S5.** Correlation matrix of mtDNA methylation at CpG positions included in the study.

## Data Availability

The raw data of mtDNA methylation are available from the corresponding author on reasonable request. SAS scripts used in the study are available upon request.

## Abbreviations

CVD: Cardiovascular disease
DBP: Diastolic blood pressure
D-loop: Non-coding position on the mtDNA
MT: Mitochondrial (standing before a name and italics denote the mitochondrially encoded genes)
*MT-CO1*: *Mitochondrially encoded cytochrome-C-oxidase I*
*MT-CO2*: *Mitochondrially encoded cytochrome-C-oxidase II*
*MT-CO3*: *Mitochondrially encoded cytochrome-C-oxidase III*
mtDNA: Mitochondrial DNA
mtDNMT: Mitochondrial DNA-methyl-transferase
*MT-OLR*: *Mitochondrially encoded light-strand-origin-of-replication*
*MT-TF*: *Mitochondrially encoded TRNA phenylalanine*
*MT-TL1*: *Mitochondrially encoded TRNA leucine 1*
SBP: Systolic blood pressure
