# Supplementary material for: Platelet mitochondrial DNA methylation predicts future cardiovascular outcome in adults with overweight and obesity
Source: Clin Epigenetics. 2020 Feb 17;12:29. doi: 10.1186/s13148-020-00825-5 (PMC7026975; doi:10.1186/s13148-020-00825-5)
Supplement: Supplementary file 1 — Additional file 1 Table S1. Multivariate analysis for the association between potential risk factors and mitochondrial DNA methylation. Table S2. Diagnostic and procedure codes for CVD events at Follow-up. Table S3. List of antihypertensive medication. Table S4. PCR primers and pyrosequencing assays used to analyze mtDNA methylation. Table S5. Correlation matrix of mtDNA methylation at CpG positions included in the study. [file 13148_2020_825_MOESM1_ESM.docx]

SUPPLEMENTARY MATERIAL

**Table S1. Multivariate analysis for the association between potential risk factors and mitochondrial DNA methylation at baseline.**

| Variable | *MT-CO1* nt6807 | | | | *MT-CO3* nt9444 | | | | *MT-TL1* nt3254 | | | |
| --- | --- | --- | --- | --- | --- | --- | --- | --- | --- | --- | --- | --- |
|  | Estimate | CI | SE | P value | Estimate | CI | SE | P value | Estimate | CI | SE | P value |
| Age | -0.001 | -0.006, 0.003 | 0.002 | 0.524 | -0.003 | -0.013,0.007 | 0.005 | 0.582 | -0.003 | -0.006, 0.000 | 0.002 | 0.083 |
| BMI | 0.001 | -0.007, 0.009 | 0.004 | 0.783 | -0.007 | -0.028, 0.013 | 0.010 | 0.492 | 0.005 | -0.002, 0.011 | 0.003 | 0.148 |
| Serum Uric Acid | 0.010 | -0.023, 0.042 | 0.016 | 0.554 | -0.024 | -0.107, 0.059 | 0.042 | 0.564 | -0.012 | -0.036, 0.012 | 0.012 | 0.330 |
| ALT | 0.000 | -0.002, 0.003 | 0.001 | 0.760 | -0.002 | -0.007, 0.002 | 0.002 | 0.334 | -0.001 | -0.002, 0.001 | 0.001 | 0.539 |
| AST | 0.001 | -0.003, 0.005 | 0.002 | 0.478 | -0.005 | -0.012, 0.002 | 0.003 | 0.133 | -0.001 | -0.004, 0.002 | 0.002 | 0.385 |
| Basophil | -1.406 | -3.819, 1.007 | 1.222 | 0.252 | -5.863 | -11.877, 0.151 | 3.005 | 0.056 | 1.238 | -0.574, 3.050 | 0.917 | 0.179 |
| Basophil percentage | -0.113 | -0.283, 0.058 | 0.086 | 0.194 | -0.475 | -0.913, -0.036 | 0.219 | **0.034** | 0.052 | -0.075, 0.179 | 0.064 | 0.419 |
| Waist circumference | 0.001 | -0.002, 0.005 | 0.002 | 0.520 | -0.005 | -0.016, 0.005 | 0.005 | 0.287 | 0.000 | -0.003, 0.003 | 0.001 | 0.941 |
| HDL cholesterol | -0.002 | -0.006, 0.001 | 0.002 | 0.170 | 0.007 | -0.003, 0.018 | 0.005 | 0.181 | 0.000 | -0.003, 0.003 | 0.001 | 0.995 |
| LDL cholesterol | -0.001 | -0.002, 0.001 | 0.001 | 0.250 | 0.000 | -0.004, 0.004 | 0.002 | 0.982 | -0.001 | -0.002, 0.001 | 0.001 | 0.326 |
| Total cholesterol | -0.001 | -0.002, 0.000 | 0.001 | 0.192 | 0.001 | -0.002, 0.004 | 0.002 | 0.628 | 0.000 | -0.001, 0.001 | 0.000 | 0.790 |
| Serum creatinine | -0.015 | -0.076, 0.045 | 0.031 | 0.620 | -0.030 | -0.135, 0.076 | 0.053 | 0.573 | -0.008 | -0.057, 0.042 | 0.025 | 0.761 |
| Eosinophils count | 0.077 | -0.287, 0.441 | 0.184 | 0.677 | -0.483 | -1.626, 0.660 | 0.571 | 0.401 | 0.118 | -0.158, 0.393 | 0.140 | 0.401 |
| Eosinophils % | 0.004 | -0.021, 0.028 | 0.012 | 0.762 | -0.039 | -0.117, 0.038 | 0.039 | 0.312 | 0.004 | -0.014, 0.023 | 0.009 | 0.635 |
| Heart Rate | 0.002 | -0.002, 0.005 | 0.002 | 0.386 | 0.000 | -0.010, 0.010 | 0.005 | 0.987 | 0.001 | -0.001, 0.004 | 0.001 | 0.333 |
| Fibrinogen | -0.001 | -0.001, 0.000 | 0.000 | **0.007** | 0.000 | -0.002, 0.002 | 0.001 | 0.991 | 0.000 | 0.000, 0.001 | 0.000 | 0.351 |
| Gamma-Glutamyltransferase | 0.000 | -0.002, 0.002 | 0.001 | 0.900 | 0.000 | -0.004, 0.005 | 0.002 | 0.857 | -0.001 | -0.002, 0.001 | 0.001 | 0.267 |
| White blood cell count | 0.010 | -0.013, 0.034 | 0.012 | 0.395 | 0.002 | -0.063, 0.067 | 0.033 | 0.957 | 0.017 | -0.001, 0.035 | 0.009 | 0.070 |
| Red blood cells count | -0.029 | -0.132, 0.073 | 0.052 | 0.574 | -0.018 | -0.261, 0.224 | 0.121 | 0.879 | 0.019 | -0.057, 0.096 | 0.039 | 0.620 |
| Granulocytes count | 0.008 | -0.021, 0.036 | 0.014 | 0.601 | -0.010 | -0.089, 0.068 | 0.039 | 0.796 | 0.020 | -0.002, 0.041 | 0.011 | 0.077 |
| Glycated haemoglobin | -0.002 | -0.009, 0.006 | 0.004 | 0.639 | -0.030 | -0.047, -0.013 | 0.008 | **0.001** | 0.005 | -0.001, 0.010 | 0.003 | 0.130 |
| Complete Blood count | 0.015 | -0.020, 0.049 | 0.017 | 0.402 | 0.028 | -0.051, 0.108 | 0.040 | 0.477 | -0.027 | -0.054, -0.001 | 0.013 | **0.041** |
| Haematocrit | 0.005 | -0.009, 0.019 | 0.007 | 0.495 | 0.010 | -0.023, 0.043 | 0.016 | 0.553 | -0.011 | -0.022, -0.001 | 0.005 | **0.038** |
| Lymphocyte count | 0.042 | -0.034, 0.118 | 0.039 | 0.279 | 0.072 | -0.113, 0.256 | 0.092 | 0.441 | 0.033 | -0.027, 0.093 | 0.030 | 0.279 |
| Lymphocyte % | 0.000 | -0.006, 0.006 | 0.003 | 0.980 | 0.007 | -0.008, 0.021 | 0.007 | 0.351 | -0.001 | -0.005, 0.003 | 0.002 | 0.671 |
| Mean Corpuscolar Volume | 0.003 | -0.003, 0.010 | 0.003 | 0.305 | 0.005 | -0.012, 0.021 | 0.008 | 0.578 | -0.006 | -0.010, -0.001 | 0.002 | **0.021** |
| Monocytes count | 0.099 | -0.131, 0.328 | 0.116 | 0.396 | 0.008 | -0.608, 0.624 | 0.308 | 0.979 | 0.000 | -0.179, 0.178 | 0.091 | 0.997 |
| Monocytes % | 0.008 | -0.015, 0.030 | 0.011 | 0.494 | -0.005 | -0.064, 0.054 | 0.030 | 0.873 | -0.011 | -0.028, 0.006 | 0.009 | 0.208 |
| Neutrophils count | 0.007 | -0.021, 0.036 | 0.015 | 0.611 | -0.007 | -0.087, 0.072 | 0.040 | 0.858 | 0.019 | -0.003, 0.041 | 0.011 | 0.089 |
| Neutrophils % | -0.001 | -0.006, 0.005 | 0.003 | 0.834 | -0.005 | -0.019, 0.010 | 0.007 | 0.524 | 0.001 | -0.003, 0.005 | 0.002 | 0.573 |
| Homocysteine | -0.003 | -0.010, 0.005 | 0.004 | 0.496 | -0.006 | -0.024, 0.013 | 0.009 | 0.538 | 0.001 | -0.005, 0.006 | 0.003 | 0.793 |
| Diastolic Blood Pressure | 0.004 | -0.003, 0.010 | 0.003 | 0.256 | -0.020 | -0.036, -0.004 | 0.008 | **0.017** | -0.003 | -0.008, 0.002 | 0.002 | 0.191 |
| Systolic Blood Pressure | -0.002 | -0.005, 0.002 | 0.002 | 0.373 | 0.012 | 0.003, 0.021 | 0.005 | **0.009** | 0.002 | 0.000, 0.005 | 0.001 | 0.095 |
| C-Reactive protein | -0.043 | -0.096, 0.010 | 0.027 | 0.113 | -0.006 | -0.152, 0.140 | 0.073 | 0.937 | 0.023 | -0.017, 0.064 | 0.020 | 0.256 |
| Platelets | 0.000 | -0.001, 0.001 | 0.000 | 0.794 | 0.000 | -0.002, 0.002 | 0.001 | 0.995 | 0.000 | 0.000, 0.001 | 0.000 | 0.196 |
| LDL/HDL | -0.079 | -0.250, 0.091 | 0.086 | 0.362 | -0.069 | -0.654, 0.516 | 0.292 | 0.815 | -0.138 | -0.265, -0.011 | 0.064 | **0.033** |
| TC/HDL | -0.020 | -0.059, 0.020 | 0.020 | 0.325 | -0.053 | -0.154, 0.048 | 0.050 | 0.297 | -0.005 | -0.036, 0.026 | 0.016 | 0.743 |
| Triglyceride | 0.001 | 0.000, 0.001 | 0.000 | 0.159 | 0.000 | -0.002, 0.003 | 0.001 | 0.711 | 0.001 | 0.000, 0.001 | 0.000 | **0.009** |
| TSH | 0.016 | -0.007, 0.038 | 0.012 | 0.181 | -0.001 | -0.049, 0.047 | 0.024 | 0.959 | 0.008 | -0.010, 0.026 | 0.009 | 0.367 |
| Waist circumference | 0.001 | -0.003, 0.004 | 0.002 | 0.625 | -0.006 | -0.016, 0.004 | 0.005 | 0.262 | -0.001 | -0.003, 0.002 | 0.001 | 0.654 |
| Neck circumference | -0.005 | -0.013, 0.003 | 0.004 | 0.222 | 0.013 | -0.003, 0.030 | 0.008 | 0.116 | -0.002 | -0.008, 0.004 | 0.003 | 0.441 |
| Inedx-HOMA-IR * | 0.000 | -0.009, 0.008 | 0.004 | 0.960 | -0.026 | -0.073, 0.020 | 0.023 | 0.259 | 0.000 | -0.006, 0.007 | 0.003 | 0.960 |
| Waist to hip ratio | 0.003 | -0.014, 0.020 | 0.009 | 0.694 | 0.076 | -0.173, 0.325 | 0.125 | 0.545 | -0.016 | -0.106, 0.073 | 0.045 | 0.722 |
| waist-to-height ratio | 0.345 | -0.253, 0.942 | 0.303 | 0.256 | -0.667 | -2.252, 0.918 | 0.792 | 0.403 | 0.309 | -0.163, 0.781 | 0.239 | 0.197 |
| Not HDL cholesterol | -0.001 | -0.002, 0.001 | 0.001 | 0.227 | 0.000 | -0.004, 0.005 | 0.002 | 0.923 | 0.000 | -0.001, 0.001 | 0.001 | 0.720 |
| HOMA-IR † | -0.001 | -0.005, 0.004 | 0.002 | 0.810 | -0.008 | -0.018, 0.001 | 0.005 | 0.080 | 0.000 | -0.004, 0.004 | 0.002 | 0.925 |
| HOMA-b ‡ | 0.000 | 0.000, 0.000 | 0.000 | 0.998 | 0.000 | -0.001, 0.000 | 0.000 | 0.343 | 0.000 | 0.000, 0.000 | 0.000 | 0.412 |
| QUICKI § | -1.382 | -4.645, 1.881 | 1.655 | 0.405 | 13.652 | 2.570, 24.735 | 5.540 | **0.017** | -1.012 | -3.434, 1.411 | 1.227 | 0.411 |
| Glucose | -0.011 | -0.041, 0.018 | 0.015 | 0.444 | 0.116 | 0.050, 0.181 | 0.033 | **0.001** | -0.027 | -0.051, -0.002 | 0.012 | **0.033** |

Logistic regression adjusted for run, batch, nuclear DNA contamination. DNA methylation variables were logtransformed. In bold are highlighted the significant P values.

*Index-HOMA-IR: Index-homeostatic model assessment (HOMA)-Insulin Resistance; † HOMA-IR: Homeostatic Model Assessment (HOMA)- Insulin Resistance; ‡HOMA-b: HOMA-beta; § QUICKI: quantitative insulin sensitivity check index

**Table S2. Diagnostic and procedure codes for CVD events at Follow-up.**

| **International Classification of Diseases, 9^th^ revision, Clinical Modifications, 2007 (ICD-9-CM-2007)** | **Frequency** | **Percentage (%)** | **Categorization mild vs severe** |
| --- | --- | --- | --- |
| 401.1 Hypertension benign | 34 | 40.48 | Mild |
| 401.9 Hypertension, Unspecified | 13 | 15.48 | Mild |
| 411.1 Intermediate coronary syndrome | 1 | 1.19 | Severe |
| 413.9 Forms of angina pectoris other than Angina decubitus and Prinzmetal Angina | 1 | 1.19 | Severe |
| 414.01 Atherosclerosis of native coronary artery | 2 | 2.38 | Severe |
| 414.8 Other specified forms of chronic ischemic heart disease | 1 | 1.19 | Severe |
| 414.9 Chronic ischemic heart disease, unspecified | 1 | 1.19 | Severe |
| 420.91 Acute idiopathic pericarditis | 1 | 1.19 | Severe |
| 423.0 Hemopericardium | 1 | 1.19 | Severe |
| 423.9 Unspecified disease of pericardium | 1 | 1.19 | Severe |
| 424.0 Mitral valve disorders | 1 | 1.19 | Severe |
| 425.1 Hypertrophic obstructive cardiomyopathy | 1 | 1.19 | Severe |
| 426.0 Atrioventricular block, third-degree | 1 | 1.19 | Severe |
| 426.3 Left bundle branch block | 1 | 1.19 | Severe |
| 427.0 Tachycardia, paroxysmal supraventricular | 1 | 1.19 | Severe |
| 427.31 Atrial fibrillation | 2 | 2.38 | Severe |
| 427.5 Cardiac arrest | 1 | 1.19 | Severe |
| 427.9 Cardiac dysrhythmia unspecified | 1 | 1.19 | Severe |
| 428.1 Left heart failure | 1 | 1.19 | Severe |
| 428.9 Heart failure, unspecified | 1 | 1.19 | Severe |
| 433.10 Occlusion and stenosis of carotid artery without mention of cerebral infarction | 1 | 1.19 | Severe |
| 436 Acute, but ill-defined, cerebrovascular disease | 1 | 1.19 | Severe |
| 437.0 Cerebral atherosclerosis | 1 | 1.19 | Severe |
| 437.1 Other generalized ischemic cerebrovascular disease | 1 | 1.19 | Severe |
| 437.3 Cerebral aneurysm, unruptured | 1 | 1.19 | Severe |
| 437.9 Unspecified cerebrovascular disease | 1 | 1.19 | Severe |
| 438.21 Late effects of cerebrovascular disease, hemiplegia affecting dominant side | 1 | 1.19 | Severe |
| 438.85 Other late effects of cerebrovascular disease, vertigo | 1 | 1.19 | Severe |
| 441.03 Abdominal aneurysm, ruptured | 1 | 1.19 | Severe |
| 441.4 Abdominal aneurysm without mention of rupture | 1 | 1.19 | Severe |
| 442.9 Aneurysm of unspecified site | 1 | 1.19 | Severe |
| 444.22 Arterial embolism and thrombosis of lower extremity | 1 | 1.19 | Severe |
| 451.19 Phlebitis and thrombophlebitis of deep veins of lower extremities, other | 2 | 2.38 | Mild |
| 455.0 Internal hemorrhoids without mention of complication | 1 | 1.19 | Mild |
| 455.5 External hemorrhoids with other complication | 1 | 1.19 | Mild |
| Stenosis | 1 | 1.19 | Severe |

The Cardiovascular diseases definition is based on 3-digits ICD-9-CM codes from 390 to 459.

**Table S3. List of antihypertensive medication.**

| **CVD-free at Follow-up (n=54 )** | | | | **CVD-developed at Follow-up (n=51)** | | | |
| --- | --- | --- | --- | --- | --- | --- | --- |
| **Pathology** | **Active ingredient** | **Frequency** | **%** | **Pathology** | **Active ingredient** | **Frequency** | **%** |
| Hypertension | Idroclortiazide | 19 | 5.81 | Hypertension | Idroclortiazide | 26 | 6.86 |
| Hypertension | Amlodipina | 10 | 3.06 | Hypertension | Enalapril | 12 | 3.17 |
| Hypertension | Enalapril | 7 | 2.14 | Hypertension | Amlodipina | 10 | 2.64 |
| Hypertension | Valsartan | 7 | 2.14 | Hypertension | Atenololo | 9 | 2.37 |
| Hypertension | Bisoprololo | 6 | 1.83 | Hypertension | Losartan | 8 | 2.11 |
| Hypertension | Perindopril | 6 | 1.83 | Hypertension | Ramipril | 7 | 1.85 |
| Hypertension | Doxazosina | 5 | 1.53 | Hypertension | Valsartan | 7 | 1.85 |
| Hypertension | Furosemide | 5 | 1.53 | Hypertension | Furosemide | 6 | 1.58 |
| Hypertension | Nebivololo | 5 | 1.53 | Hypertension | Olmesartan medoximil | 6 | 1.58 |
| Hypertension | Ramipril | 5 | 1.53 | Hypertension | Doxazosina | 5 | 1.32 |
| Hypertension | Atenololo | 4 | 1.22 | Hypertension | Nebivololo | 4 | 1.06 |
| Hypertension | Olmesartan medoximil | 4 | 1.22 | Hypertension | Amiloride | 4 | 1.06 |
| Hypertension | Irbesartan | 3 | 0.92 | Hypertension | Bisoprololo | 3 | 0.79 |
| Hypertension | Lercanidipina | 3 | 0.92 | Hypertension | Irbesartan | 3 | 0.79 |
| Hypertension | Telmisartan | 3 | 0.92 | Hypertension | Telmisartan | 3 | 0.79 |
| Hypertension | Amiloride | 2 | 0.61 | Hypertension | Lisinopril | 2 | 0.53 |
| Hypertension | Barnipidina | 2 | 0.61 | Hypertension | Acido acetilsalicilico | 2 | 0.53 |
| Hypertension | Candesartan | 2 | 0.61 | Hypertension | Carvedilolo | 2 | 0.53 |
| Hypertension | Carvedilolo | 2 | 0.61 | Hypertension | Diltiazem | 2 | 0.53 |
| Hypertension | Lacidipina | 2 | 0.61 | Hypertension | Verapamil | 2 | 0.53 |
| Hypertension | Lisinopril | 2 | 0.61 | Hypertension | Candesartan | 2 | 0.53 |
| Hypertension | Losartan | 2 | 0.61 | Hypertension | Olmesartan medoximil | 1 | 0.26 |
| Hypertension | Manidipina | 2 | 0.61 | Hypertension | Perindopril | 1 | 0.26 |
| Hypertension | Nifedipina | 2 | 0.61 | Hypertension | Quinapril | 1 | 0.26 |
| Hypertension | Zofenopril | 2 | 0.61 | Hypertension | Clonidina | 1 | 0.26 |
| Hypertension | Barnidipina | 1 | 0.31 | Hypertension | Barnipidina | 1 | 0.26 |
| Hypertension | Clonidina | 1 | 0.31 | Hypertension | Felodipina | 1 | 0.26 |
| Hypertension | Clortalidone | 1 | 0.31 | Hypertension | Lercanidipina | 1 | 0.26 |
| Hypertension | Fosinopril | 1 | 0.31 | Hypertension | Manidipina | 1 | 0.26 |
| Hypertension | Indapamide | 1 | 0.31 | Hypertension | Nifedipina | 1 | 0.26 |
| Hypertension | Metoprololo | 1 | 0.31 | Hypertension | Canrenone | 1 | 0.26 |
| Hypertension | Olmesartan medoximil | 1 | 0.31 | Hypertension | Clortalidone | 1 | 0.26 |
| Hypertension | Piretanide | 1 | 0.31 | Hypertension | Aliskiren | 1 | 0.26 |
| Hypertension | Propranololo | 1 | 0.31 | Hypertension | Rosuvastatina | 1 | 0.26 |
| Hypertension | Quinapril | 1 | 0.31 |  |  |  |  |
| Hypertension | Spironolattone | 1 | 0.31 |  |  |  |  |

This table shows the antihypertensive drugs used at Baseline by the participants. The medication usage is stratified between CVD-free and CVD-developed at Follow-up. Antihypertensive medications represent the 36.38% of the total drugs used by the CVD-free at Follow-up and 37.64% of the total drugs used by the CVD-developed at Follow-up.

**Table S4. PCR primers and Pyrosequencing assays used to analyze mtDNA methylation.**

| Assay | Primer sequence | Measured CpG (nt#)* | Product size (bp) | Annealing temperature (°C) |
| --- | --- | --- | --- | --- |
| *MT-CO1* | Forward: TATTAATTGGTTTTTTAGGGTTTAT  Reverse-biotin labelled: CAACAAATCATTTCATATTACTTCC  Sequencing primer: TATTTATAGTAGGAAT  Sequencing entry: AGAC/tGTAGATATAC/tGAGTATATTTTATTTT | 6797  6807 | 177 | 52 |
| *MT-CO2* | Forward: TTTATGAGTTGTTTTTATATTAGGTTTAAA  Reverse-biotin labelled: ACTCCACAAATTTCAAAACATTAAC  Sequencing primer: TAAAAATAGATGTAAT  Sequencing entry: TTTC/tGGAC/tGTTTAAATTAAA | 8113  8117 | 123 | 52 |
| *MT-CO3* | Forward: TATATTATTTGTTTAAAAAGGTTTT  Reverse-biotin labelled: AATAAAAAACTCAAAAAAATCCTAC  Sequencing primer: TATATTATTTGTTTAAAAAGGTTTT  Sequencing entry: CGATAYGGGATAATTTTATT | 9444  9449 | 95 | 52 |
| *MT-TL1* | Forward: TAGGGTTTGTTAAGATGGTAGAGTT  Reverse-biotin labelled: ACAATAAAAAATAAAAAATTAACCATAAAT  Sequencing primer: TAGGGTTTGTTAAGATGGTAGAGTT  Sequencing entry: TAGGGTTTGTTAAGATGGTAGAGTT | 3247  3254 | 117 | 52 |
| D-loop | Forward: TGTGTAGATATTTAATTGTTATTA  Reverse-biotin labelled: CAAATCTATCACCCTATTAACCAC  Sequencing primer: TAATTAATTAATATATTT  Sequencing entry: TAGTAAATATGTTC/tGTTT | 16383 | 254 | 52 |
| *MT-TF* | Forward: TAAAGTAATATATTGAAAATGTTTAGA  Reverse-biotin labelled: TACTTAATACTTATCCCTTTTAAT  Sequencing primer: TATTGAAAATGTTTA  Sequencing entry: GAC/tGGGTTTATATTA | 624 | 168 | 52 |
| *MT-OLR* | Forward: AATTGGTTTTAATTTATTTTTTT  Reverse-biotin labelled: AACCTCTTTTTACCAACTCC  Sequencing primer: AATTGGTTTTAATTTATTTTTTT  Sequencing entry: C/tGTC/tGTC/tGGGAAAAAAGGT | 5737  5740  5743 | 126 | 52 |

*nt stands for “nucleotide” and represent the position of the CpG site based on the mtDNA sequence “NC_012920.1”.

**Table S5. Correlation matrix of mtDNA methylation at CpG position included in the study.**

| Position  (nt#) | *MT-CO1* 6797 | *MT-CO1* 6807 | *MT-CO2* 8113 | *MT-CO2*  8117 | *MT-CO3* 9444 | *MT-CO3* 9449 | *MT-TL1* 3247 | *MT-TL1* 3254 | D-loop 16383 | *MT-TF* 624 | *MT-OLR* 5737 | *MT-OLR*  5740 | *MT-OLR*  5743 |
| --- | --- | --- | --- | --- | --- | --- | --- | --- | --- | --- | --- | --- | --- |
| *MT-CO1* 6797 | 1 | 0.77647 | 0.1761 | 0.10858 | -0.10996 | -0.09612 | -0.14134 | 0.22877 | 0.46573 | 0.01773 | 0.08875 | 0.29655 | 0.33849 |
|  |  | <.0001 | 0.0146 | 0.1349 | 0.1269 | 0.1836 | 0.0482 | 0.0013 | <.0001 | 0.8052 | 0.2149 | <.0001 | <.0001 |
| *MT-CO1* 6807 | 0.77647 | 1 | 0.1618 | 0.13884 | 0.04579 | 0.01438 | -0.15348 | 0.28669 | 0.50499 | 0.06199 | 0.15897 | 0.34708 | 0.36526 |
|  | <.0001 |  | 0.025 | 0.0554 | 0.5261 | 0.8426 | 0.0317 | <.0001 | <.0001 | 0.3881 | 0.0257 | <.0001 | <.0001 |
| *MT-CO2* 8113 | 0.1761 | 0.1618 | 1 | 0.35736 | 0.24829 | 0.20411 | 0.35101 | 0.05281 | 0.16581 | 0.46865 | 0.34074 | 0.20646 | 0.14159 |
|  | 0.0146 | 0.025 |  | <.0001 | 0.0005 | 0.0046 | <.0001 | 0.4646 | 0.0257 | <.0001 | <.0001 | 0.0038 | 0.0489 |
| *MT-CO2*  8117 | 0.10858 | 0.13884 | 0.35736 | 1 | 0.01637 | 0.11327 | 0.0784 | 0.10325 | 0.21243 | 0.18628 | 0.1935 | 0.29934 | 0.22649 |
|  | 0.1349 | 0.0554 | <.0001 |  | 0.8222 | 0.1197 | 0.2785 | 0.153 | 0.0042 | 0.0095 | 0.0069 | <.0001 | 0.0015 |
| *MT-CO3* 9444 | -0.10996 | 0.04579 | 0.24829 | 0.01637 | 1 | 0.55779 | 0.42585 | 0.04138 | -0.11953 | 0.41703 | 0.55029 | 0.18602 | 0.14008 |
|  | 0.1269 | 0.5261 | 0.0005 | 0.8222 |  | <.0001 | <.0001 | 0.5647 | 0.1061 | <.0001 | <.0001 | 0.0089 | 0.0502 |
| *MT-CO3* 9449 | -0.09612 | 0.01438 | 0.20411 | 0.11327 | 0.55779 | 1 | 0.16685 | -0.01355 | -0.03266 | 0.21639 | 0.34343 | 0.11986 | 0.13468 |
|  | 0.1836 | 0.8426 | 0.0046 | 0.1197 | <.0001 |  | 0.0197 | 0.8509 | 0.6608 | 0.0024 | <.0001 | 0.0943 | 0.0605 |
| *MT-TL1* 3247 | -0.14134 | -0.15348 | 0.35101 | 0.0784 | 0.42585 | 0.16685 | 1 | 0.08669 | -0.14277 | 0.31831 | 0.38255 | 0.22996 | 0.15047 |
|  | 0.0482 | 0.0317 | <.0001 | 0.2785 | <.0001 | 0.0197 |  | 0.2234 | 0.0525 | <.0001 | <.0001 | 0.0011 | 0.0343 |
| *MT-TL1* 3254 | 0.22877 | 0.28669 | 0.05281 | 0.10325 | 0.04138 | -0.01355 | 0.08669 | 1 | 0.29298 | 0.14493 | 0.13576 | 0.30944 | 0.39609 |
|  | 0.0013 | <.0001 | 0.4646 | 0.153 | 0.5647 | 0.8509 | 0.2234 |  | <.0001 | 0.0416 | 0.0559 | <.0001 | <.0001 |
| D-loop 6383 | 0.46573 | 0.50499 | 0.16581 | 0.21243 | -0.11953 | -0.03266 | -0.14277 | 0.29298 | 1 | 0.05739 | 0.28847 | 0.32449 | 0.40303 |
|  | <.0001 | <.0001 | 0.0257 | 0.0042 | 0.1061 | 0.6608 | 0.0525 | <.0001 |  | 0.4378 | <.0001 | <.0001 | <.0001 |
| *MT-TF* 624 | 0.01773 | 0.06199 | 0.46865 | 0.18628 | 0.41703 | 0.21639 | 0.31831 | 0.14493 | 0.05739 | 1 | 0.30585 | 0.05395 | 0.06999 |
|  | 0.8052 | 0.3881 | <.0001 | 0.0095 | <.0001 | 0.0024 | <.0001 | 0.0416 | 0.4378 |  | <.0001 | 0.4492 | 0.3272 |
| *MT-OLR* 5737 | 0.08875 | 0.15897 | 0.34074 | 0.1935 | 0.55029 | 0.34343 | 0.38255 | 0.13576 | 0.28847 | 0.30585 | 1 | 0.5669 | 0.54853 |
|  | 0.2149 | 0.0257 | <.0001 | 0.0069 | <.0001 | <.0001 | <.0001 | 0.0559 | <.0001 | <.0001 |  | <.0001 | <.0001 |
| *MT-OLR* 5740 | 0.29655 | 0.34708 | 0.20646 | 0.29934 | 0.18602 | 0.11986 | 0.22996 | 0.30944 | 0.32449 | 0.05395 | 0.5669 | 1 | 0.81336 |
|  | <.0001 | <.0001 | 0.0038 | <.0001 | 0.0089 | 0.0943 | 0.0011 | <.0001 | <.0001 | 0.4492 | <.0001 |  | <.0001 |
| *MT-OLR*  5743 | 0.33849 | 0.36526 | 0.14159 | 0.22649 | 0.14008 | 0.13468 | 0.15047 | 0.39609 | 0.40303 | 0.06999 | 0.54853 | 0.81336 | 1 |
|  | <.0001 | <.0001 | 0.0489 | 0.0015 | 0.0502 | 0.0605 | 0.0343 | <.0001 | <.0001 | 0.3272 | <.0001 | <.0001 |  |

Correlation size and interpretation: Very high positive (negative) correlation 0.90 to 1.00 (−0.90 to −1.00); High positive (negative) correlation 0.70 to 0.90 (−0.70 to −0.90); Moderate positive (negative) correlation 0.50 to 0.70 (−0.50 to −0.70); Negligible correlation 0.30 to 0.50 (−0.30 to −0.50); Negligible correlation 0.00 to 0.30 (0.00 to −0.30). “nt” stands for “nucleotide” position and it is based on the mtDNA sequence “NC_012920.1”.
